# Advanced fabrication and characterization of thin-film composite polyamide membranes for superior performance in reverse osmosis desalination

**DOI:** 10.1038/s41598-025-97871-x

**Published:** 2025-04-30

**Authors:** Ayman Eltahan, Nesreen Ismail, Marwa Khalil, Shaker Ebrahim, Moataz Soliman, Ehssan Nassef, Ashraf Morsy

**Affiliations:** 1 Department of Physics, Faculty of Science, Tanta University, Tanta, Egypt; 2 Alexandria Water Company, Alexandria, Egypt; 3 Composites and Nano Structured Materials Research Dept., Advanced Technology and New Materials Research Institute (ATNMRI), City of Scientific Research and Technological Applications (SRTA-City), New Borg El-Arab City, P.O. Box: 21934, Alexandria, Egypt; 4 Materials Science Department, Institute of Graduate Studies and Research, Alexandria University, 163 Horreya Avenue, El-Shatby, PO Box 832, Alexandria, Egypt; 5 Department of Petrochemicals, Faculty of Engineering, Pharos University in Alexandria; Canal El Mahmoudia Street, Beside Green Plaza Complex 21648, Alexandria, Egypt

**Keywords:** Thin film, Composite membranes, Polyamide, Reverse osmosis, Water desalination, Environmental sciences, Chemistry, Energy science and technology, Engineering, Materials science

## Abstract

Thin film composite (TFC) polyamide membranes are crucial for efficient reverse osmosis (RO) desalination, offering high selectivity and permeability. This study investigates the fabrication and optimization of TFC membranes on polysulfone supports, focusing on their structural, morphological, and performance properties for enhanced desalination efficiency using the phase inversion technique, a method that enables precise control over membrane structure. Key fabrication parameters including the concentrations of m-phenylene diamine (MPD) and trimesoyl chloride (TMC), and the immersion times for both monomers were systematically varied to investigate their impact on membrane hydrophilicity, morphology, and structure. Hydrophilicity was assessed via contact angle measurements, Scanning electron microscopy was used to characterize the morphology (SEM), and structural properties were analyzed by Fourier-transform infrared spectroscopy (FTIR). The RO membranes’ desalination performance was evaluated by measuring water flux and salt rejection in a cross-flow setup with saline water (10,000 ppm) under controlled processing conditions. Results indicated that variations in MPD and TMC concentrations, as well as immersion times, significantly influenced membrane hydrophilicity and pore structure, affecting water flux and salt rejection. The maximum salt rejection and water flux for the prepared thin film composite reverse osmosis membrane were 98.6% and 19.1 L/m^2^ h, respectively obtained at m-phenylenediamine concentration of 2 wt% and tri mesoyl chloride concentration of 0.1 wt/v reacted for 1 min. The study provides insights into optimizing TFC-RO membrane fabrication parameters to enhance desalination efficiency, highlighting the potential of these membranes for high-performance RO desalination applications.

## Introduction

In recognition of their important role in desalination and water purification, thin-film composite (TFC) membranes have seen remarkable growth in the area. Much research has been done on improving preparation conditions to improve membrane performance. TFC membranes’ excellent salt rejection and water flow capabilities are largely due to the action^1^. The key component of TFC membranes’ high salt rejection and water flow capacities is the active polyamide layer, which is created via the interfacial polymerization (IP) approach on a porous support^2^. The Shotten–Bauman reaction between an amine and an acid chloride frequently yields polyamides, which provide the required structural properties that allow TFC membranes to outperform other kinds in terms of permeability and selectivity^3–6^.

Due to limited acid chloride solubility in water, the IP reaction usually takes place between monomers in two immiscible phases, where a diamine in an aqueous solution diffuses into an organic solution containing a dichloride, creating the polyamide layer on the organic phase side of the interface^7,8^. The concentration of monomers and the concentration ratio affect the structure of polyamides, which include both linear and cross-linked sections with free carboxylic acid groups^9,10^. A crucial component of TFC membrane applications, its structure regulates the membrane’s mechanical stability in addition to its chemical characteristics^11–13^. Depending on the reaction conditions, the resultant film can have a thickness of 10 nm to several microns. The IP process, which takes place at the organic interface, is quick and frequently self-limiting^14–17^. When creating high-performance TFC membranes, the degree of cross-linking is crucial since it influences not only the membranes’ mechanical qualities but also their rejection and permeability^18–20^.

Research has demonstrated that different monomer types, like MPD with TMC, can result in improved performance because of the additional cross-linking potential. Other monomer types, such as 3,4,5-biphenyl triacyl chloride, add hydrophilicity, though occasionally at the expense of water flux^21,22^. Monomer concentration and duration are also important; studies have shown that TMC concentration and lag time have a direct impact on membrane thickness, surface hydrophilicity, and performance metrics including flow and salt rejection^23,24^. Increased TMC concentration results in enhanced hydrophilicity, which affects the interfacial energy and improves water flux even more^25^. The inhomogeneous charge distribution and dense core structure of polyamide films generated by IP are revealed by molecular simulations, which are employed in addition to experimental methods to investigate the dynamics of IP at the molecular level^26^. Findings showing the important role of monomer diffusion and the fast self-limiting nature of the polymerization process, which influence the rate and degree of cross-linking in the active layer, further support the association between preparation conditions and membrane morphology^27–29^. This study aims to optimize the synthesis of TFC membranes through controlled variations in monomer concentrations and reaction conditions to achieve an optimal balance between water flux and salt rejection. Unlike previous research, which primarily focused on single-variable modifications, this work systematically investigates the combined effects of monomer ratios, reaction times, and interfacial conditions on membrane structure and performance. By enhancing the fundamental understanding of TFC membrane formation and its influence on separation efficiency, this study seeks to advance membrane technology for more efficient desalination and water purification processes.

## Materials and methods

### Materials

Materials from Acros Company were used in this work, including 1,3,5-benzene tricarbonyl trichloride with a purity of 98%, *m*-phenylene diamine with a purity of 99% or higher, and polysulfone pellets with a molecular weight of 60,000. The TEDIA Company provided the 95% *n*-hexane, and Fisher Company supplied the *N*-methyl pyrrolidone. Furthermore, MP Biomedical supplied sodium chloride (NaCl).

### Methods

#### Polysulfone membrane preparation

The polysulfone support membrane (PS) is made by 18 g of PSF (MW = 60,000) was thoroughly dissolved in 72 mL of *N*-methyl pyrrolidone (NMP) while being vigorously stirred. To get rid of air bubbles, the solution was left to settle overnight. After that, it was cast using an automatic film applicator with a 250 µm thickness on a glass plate. Until the PSF membrane split from the glass plate, the PSF-covered glass plate was submerged in deionized water (a non-solvent) at room temperature. Deionized water was used to wash the membrane and to preserve it for interfacial polymerization.

### Thin film composite membrane

Five series of membranes (I, II, III, IV and V) were prepared with varying conditions as shown in Table 1

**Table 1:** Different ratio of monomer.

Sample	MPD (wt %)	MPD soaking time (min)	TMC (wt %)	TMC reaction time (min)
I	0.5	2	0.1	1
II	1	2	0.1	1
III	1.5	2	0.1	1
IV	2	2	0.1	1
V	2.5	2	0.1	1

### Variation of MPD concentration during IP of polyamide

**Fig. 1:**
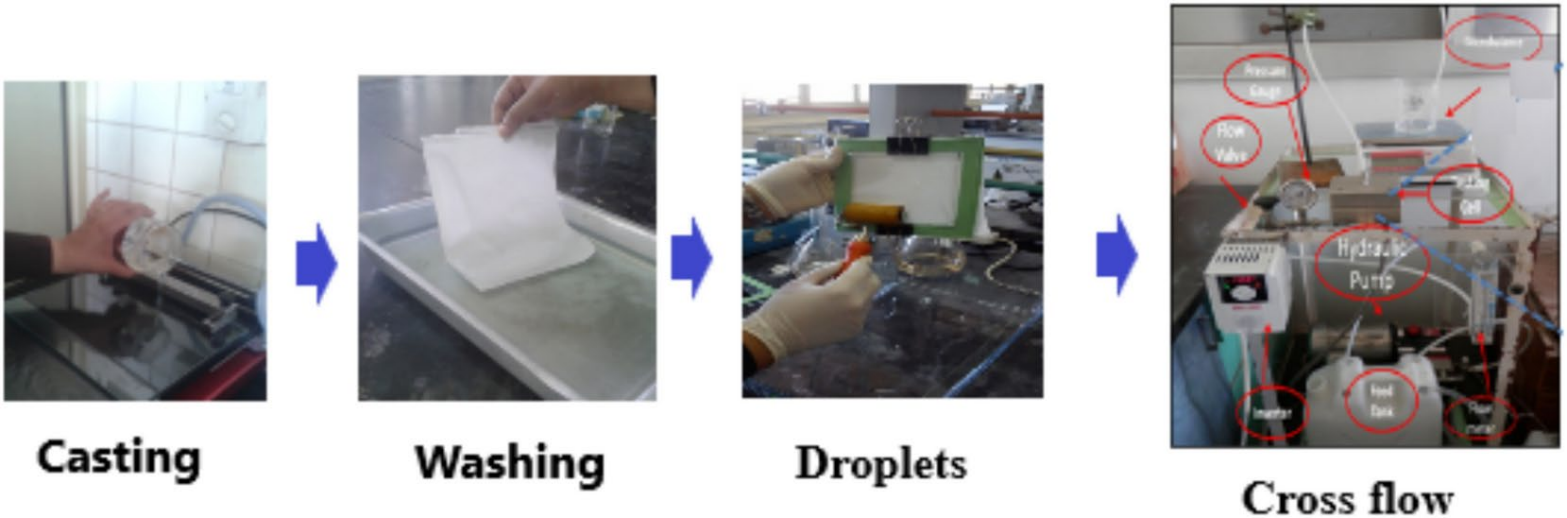
Schematic diagram of the membrane experimental setup.

After spending the night submerged in deionized water, a PSf support membrane was placed on a plastic plate. On top was a plastic frame that was secured with clips. Different concentrations were utilized, enabling contact for two min (0.5, 1, 1.5, 2, 2.5 g MPD/100 mL water). After removing the remaining solution, the membrane was rinsed with 100 mL of *n*-hexane, cured for 5 min at 75 °C in an oven, and then stored for the night in a dry, dark location as shown in Fig. 1.

### Characterization

The chemical structure of the membranes was identified using the Fourier transform infrared (FTIR) technique (Spectrum BX 11 Infrared Spectrometer FTIR LX 18-5255 Perkin Elmer). For the TFC-RO membranes, the spectra were acquired in the 4000–400 cm^−1^ wavenumber region. The CA samples were prepared by grinding KBr and CA at a 10:1 ratio. As thin films, the RO membranes underwent testing. Using the scanning electron microscope (SEM) (XL 30 JEOL), The TFC-RO membranes’ morphology was examined by examining the membranes’ surface, bottom, and cross section. Using a contact angle metre (Rame hart, Instrument was used to determine the contact angle. The performance (salt rejection and water flux) of the TFC-RO membranes (area 42 cm^2^) was evaluated using the cross flow RO unit (CF042, Sterlitech, USA) with hydra pump, pressure control valve, and gauge through rejection line, variable frequency drive (SV015IG5A-4), and flow metre F-550 (USA) (2). Permeate flux and salt rejection were measured using cross flow filtration and aqueous feed solution containing 10,000 ppm NaCl.

## Results and discussion

### Structure of polysulfone and TFC membranes

**Fig. 2:**
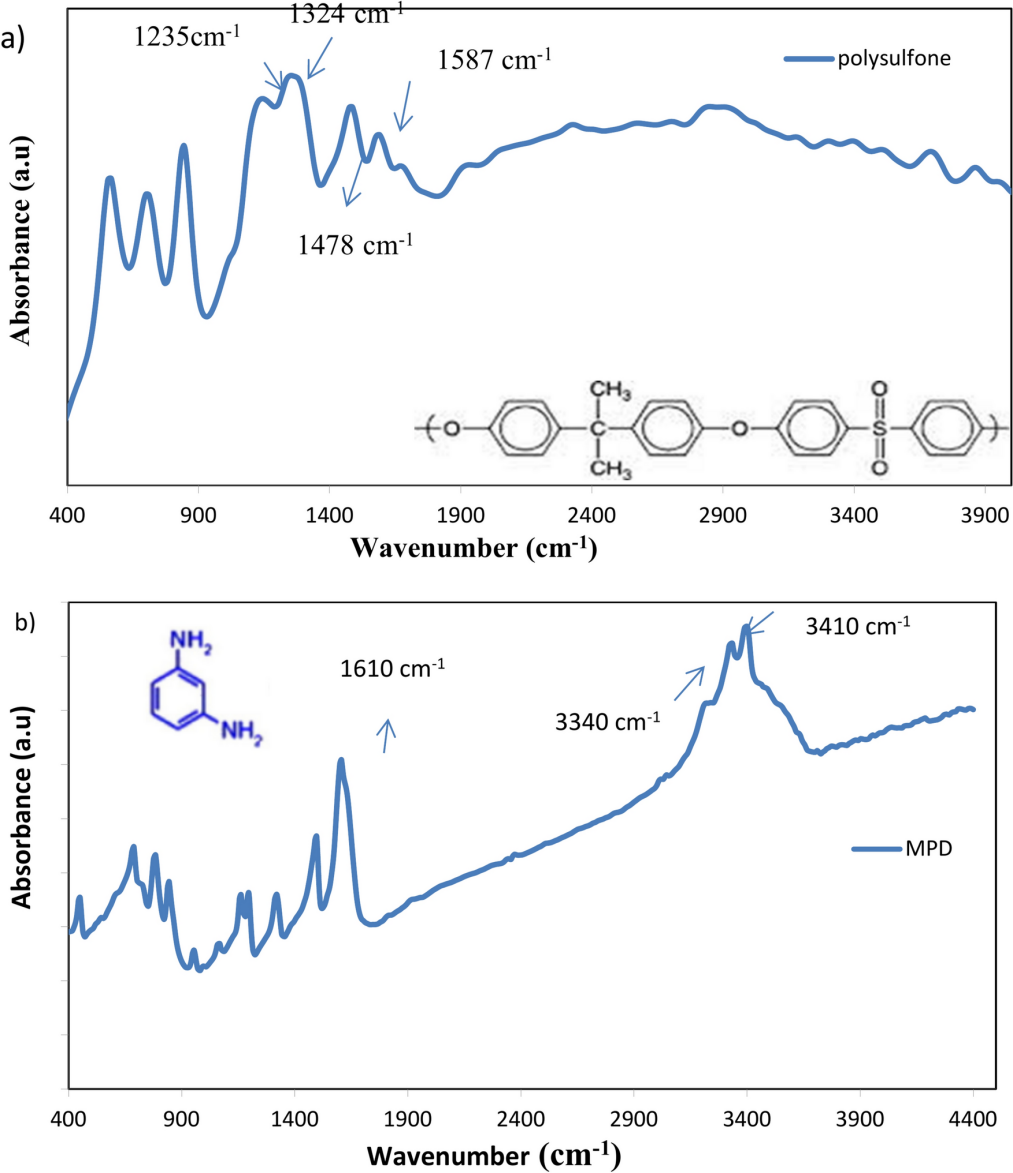

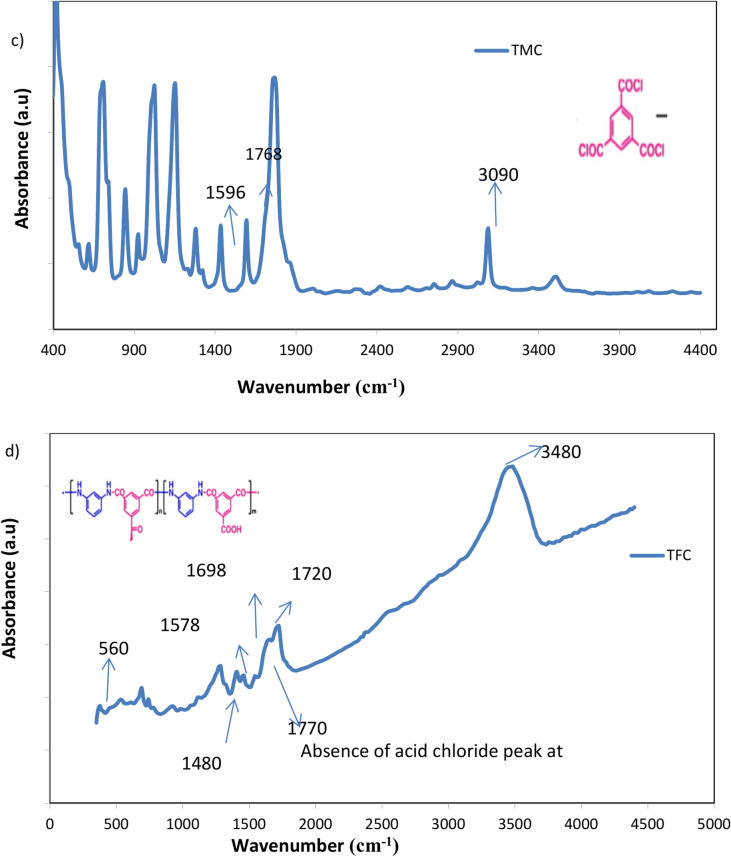
FTIR of (**a**) PS support layer, (**b**) MPD, (**C**) TMC and (**d**) PS/PA-TFC membrane.

Figure 1 presents the FTIR analysis of the chemical structure of the PA-TFC membrane surface and the PSf support layer. The PSf membrane spectrum Fig. 2a exhibits peaks at 1587, 1487, 1324, 1294, 1235, and 1150–1106 cm⁻^1^, corresponding to C–C, C–H, symmetric O=S=O, C–O–C, and asymmetric O=S=O, respectively, indicating the presence of both the polyamide barrier layer and the underlying PSf substrate. Peaks at 1610, 3410, and 3440 cm^−1^ in the MPD monomer spectrum Fig. 2b are attributed to the benzene ring’s C–C, the sym NH, and the anti-symmetric NH bond, respectively. Peaks at 1596, 1768, and 3090 cm^−1^ in the TMC monomer spectrum Fig. 2c are attributed to the benzene ring’s C–C, C–O, and C–H. A successful polymerization has taken place, as evidenced by the disappearance of the acid chloride band at 1770 cm^−1^ in the PA-TFC membrane spectra Fig. 2d^30^. The bands at 1698 cm⁻^1^ and 1549 cm⁻^1^ correspond to the amide I (C=O stretching) and amide II (N–H bending) vibrations of the amide group (–CONH–), respectively. Additionally, there were additional PA-specific bands at 1250 cm^−1^ (amide III) and 1610 and 1489 cm^−1^ (aromatic ring breathing). Additionally, the OH group’s stretching peak is located at 3480 cm^−1^.

### The hydrophilic properties of TFC membranes

**Fig. 3:**
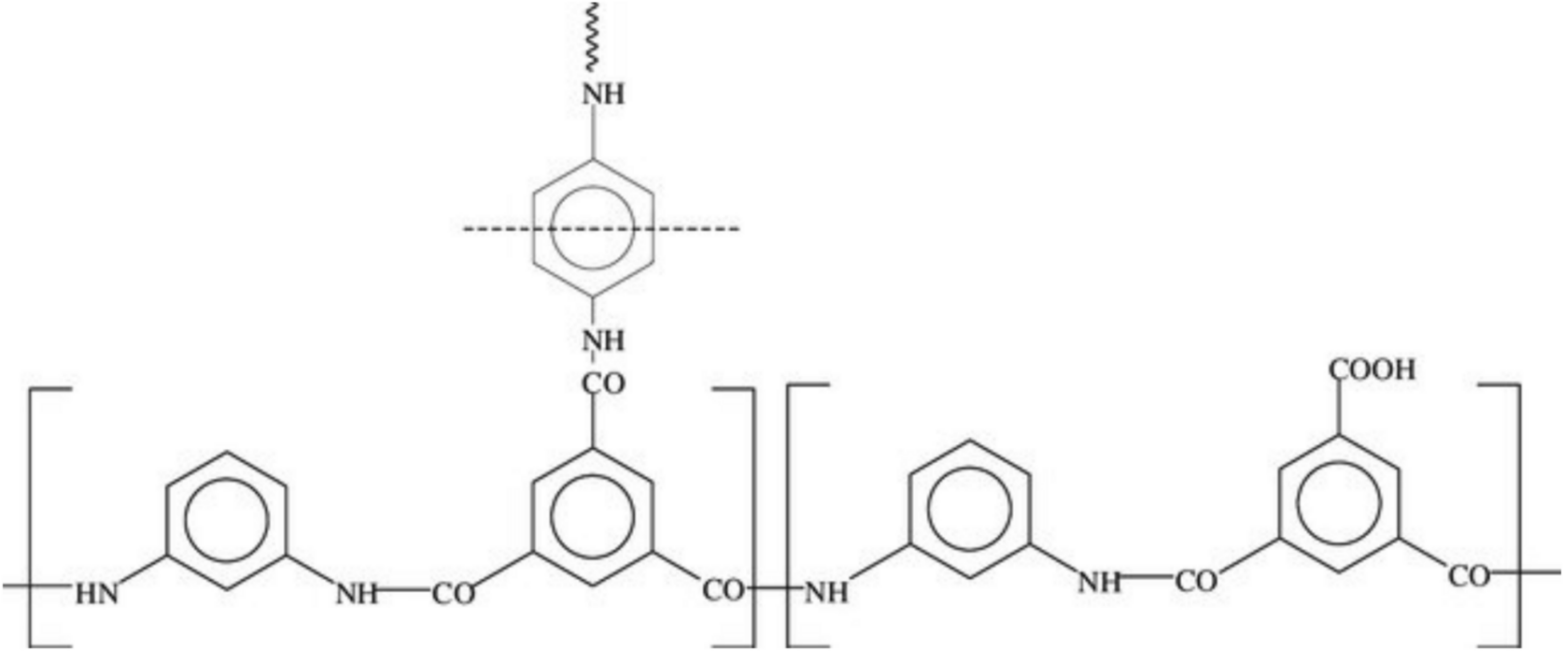
Polyamide structure resulting from the interfacial polymerization of MPD and TMC.

The hydrophilic characteristic is connected with the membrane’s surface energy and is an intriguing property to measure. Because of this, when working with membrane fouling, surface energy and surface roughness are two of the most intriguing characteristics to take into account. This is particularly true if the solution to be filtered contains proteins and other physically active materials^31^. Contact angle measurements can be used to assess the manufactured TFC RO membranes’ surface hydrophilicity. A significant observation is the decrease in contact angle, indicating an increase in hydrophilicity. The contact angle drops from approximately 70° for pure polyamide to around 40° for the nanocomposite with the highest nanoparticle loading. Water filtration applications often benefit from enhanced water permeability, salt rejection, and fouling resistance when the film layer is moderately smooth, hydrophilic, and negatively charged^32^. Because the PA layer (Fig. 3) on top of the PS microporous support layer contains amide, carboxylic, and hydroxyl functional groups that increase surface hydrophilicity and surface free energy, the contact angle of the polysulfone membrane is found to be 91°. Therefore, the PA layer reduces the contact angle.

**Fig. 4:**
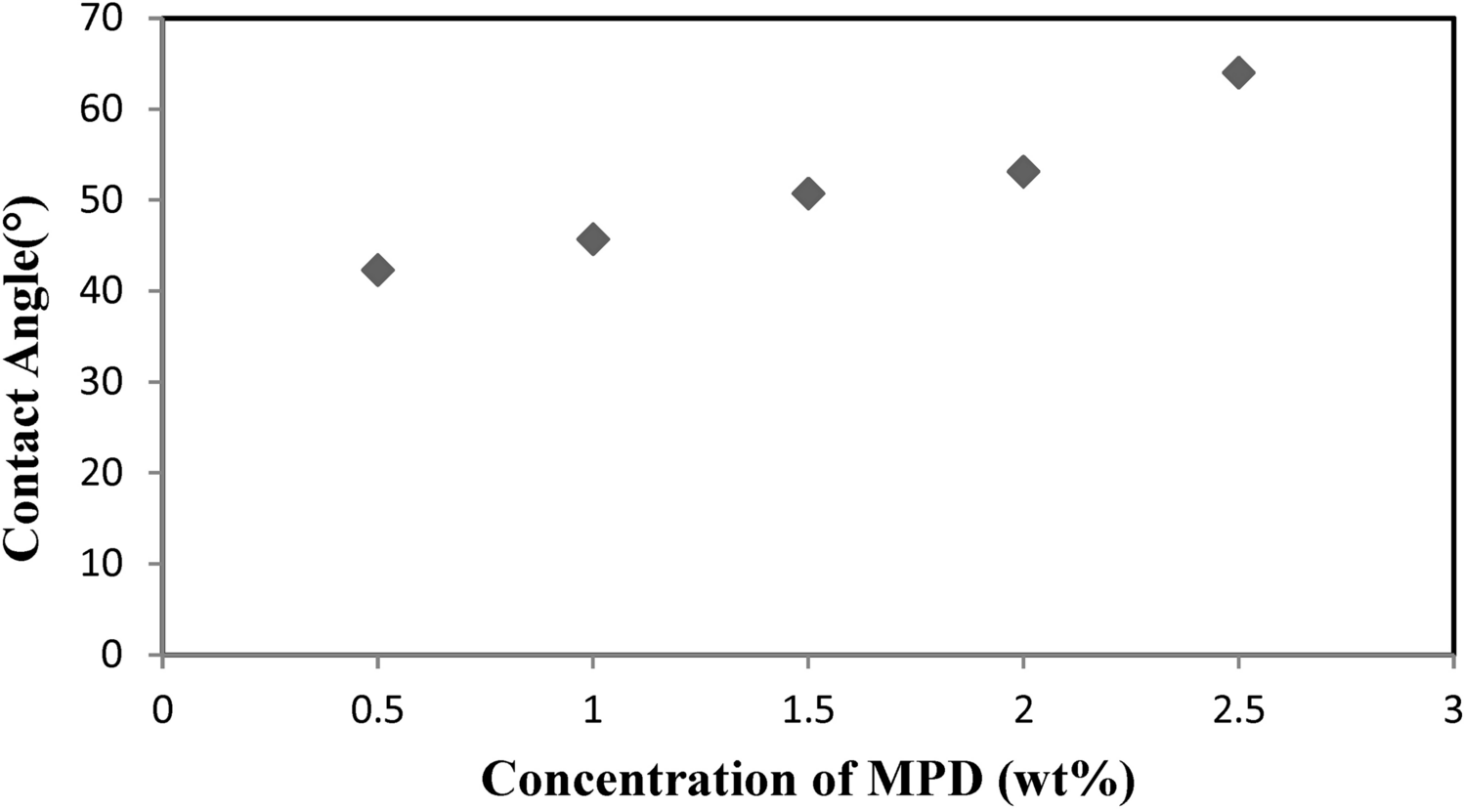
Contact angle of TFC-RO membrane prepared with different concentration of MPD soaking time (2 min) and 0.1 wt% TMC.

Figure 4 illustrates how the concentration of MPD affects the contact angles of TFC-RO membranes made with varying MPD concentrations, MPD soaking times of two minutes, and TMC 0.1w/v% immersion times of one minute. It has been seen that when the MPD concentration has increased, the contact angle has gradually increased (i.e., hydrophilicity has decreased).

Figure 2 displays the X and Y fractions as well as the repetition unit’s chemical formula. The Y-fraction behavior suggests that the active layer polymerized at low TMC concentrations is less hydrophilic than that formed at higher TMC concentrations. Water solubility, influenced by both hydrophilicity and Y-fraction, increases as the TMC concentration rises^33^. As the condensation reaction between amine and acid chloride occurs at the aqueous-organic interface, the chemical composition of the active layer reflects the unique characteristics of the interfacial polymerization (IP) process. Consequently, the diffusion of MPD through the polymerized film and TMC diffusion in the organic phase play a significant role in the reaction^34^. The TMC concentration is crucial in determining the active layer’s properties when polymerization is controlled by TMC diffusion. As expected, the thickness of the thin film increases linearly with TMC concentration. With a relatively high MPD concentration, the likelihood of unreacted acid chloride groups decreases as TMC concentration drops, making it easier to hydrate any remaining acid chloride groups to form carboxylic acids^35^. However, as Fig. 3 illustrates, the hydrophilicity is dependent on the MPD concentration. As the concentration of MPD increased it also raised the contact angle. The water solubility reduced as the MPD concentration increased, and the membrane surface is more hydrophilic at low MPD concentrations than those generated at high MPD concentrations. Figure 3 illustrates that as the MPD concentration increased from 0.5 to 2.5 wt/v%, the TMC concentration remained constant at 0.1 wt/v%. The likelihood of unreacted acid chloride groups decreases with higher MPD concentration when polymerization is controlled by MPD diffusion, which may explain the observed increase in contact angle and decrease in hydrophilicity. According to most theoretical simulations of the process, the diffusion of the aqueous-phase monomer through the newly-formed polyamide layer limits its growth rate. These models suggest that the thickness of the polyamide layer scales with √t (where t is the polymerization time), and the growth rate of the polyamide layer follows 1/√t if the diffusion constant of the aqueous-phase monomer in polyamide remains constant. However, since the density of the polyamide layer is assumed to increase over time, the assumption of a time-independent diffusion constant is not strictly accurate. This has led to the development of multi-stage models^36^.

This is a result of polyamide’s enhanced amide bond and free carboxylic acid group concentration, which accelerated MPD’s diffusion into the organic phase. This is brought on by a rise in film thickness, surface roughness, and the solid–liquid interface. Extending the immersion duration of TMC or MPD leads to the formation of a linear section of PA with free –COOH groups, resulting in a decrease in the contact angle of the PA-TFC membranes and an increase in hydrophilicity^37^. TFC-RO membranes have a same rough surface; hence the polymerization time has little effect on the water contact angle.

### Morphology of the membrane surface

**Fig. 5:**
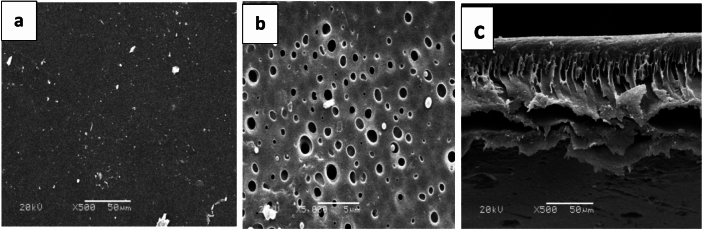
SEM images of polysulfone for top surface (**a**), bottom (**b**), and cross section (**c**).

**Fig. 6:**
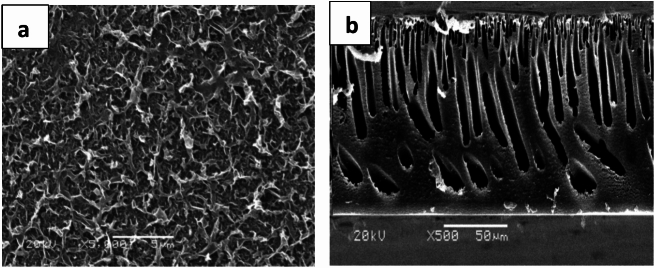
SEM images of PA-TFC membranes showing the top surface (**a**) and the cross-section (**b**).

Figures 5 and 6 display the SEM images of the membrane surfaces and cross-sections. The synthesized polyamide TFC surface exhibited tightly packed globules and scattered, ear-shaped polyamide ridges. These structures were likely formed on top of the polysulfone support layer with surface pores, allowing MPD to diffuse into the organic phase and create these finger-like formations. In contrast, the PSf porous layer has a smooth surface and a porous bottom. These figures show that every membrane has an asymmetric structure with a porous sublayer and a dense top layer. Both macro void structure and finger-like cavities are present in the sublayers. Instantaneous demixing occurs as a result of the solvent’s high mutual attraction for water, which causes finger-like cavities to form in the prepared membranes’ sublayer^38^.

The top surface may have formed as a result of the casting solution demixing through the nucleation and development of the solid phase, which is the polymer-rich phase. Better linked pores result from the surface nodule/aggregate development. Membrane morphology may be impacted by any of the two forms of demixing that might occur during the phase inversion process: immediate demixing or delayed demixing. Instantaneous demixing membranes typically exhibit a thin, porous skin layer and a highly porous substructure with macro spaces. Membranes with a dense, slightly thick skin layer and a porous (typically closed-cell, macro void-free) substructure are produced by a delayed demixing mechanism. The structure and properties of membranes produced by the phase inversion process are influenced by factors such as the choice of solvent or non-solvent, annealing temperature, molecular weight, additive type, and other variables. These parameters can either affect the macro void’s expansion or suppression. While some of these actors have the propensity to create macro voids, others aid in their suppression, enhancing pore interconnectivity and raising porosities in both the top and sublayers^39,40^.

**Fig. 7:**
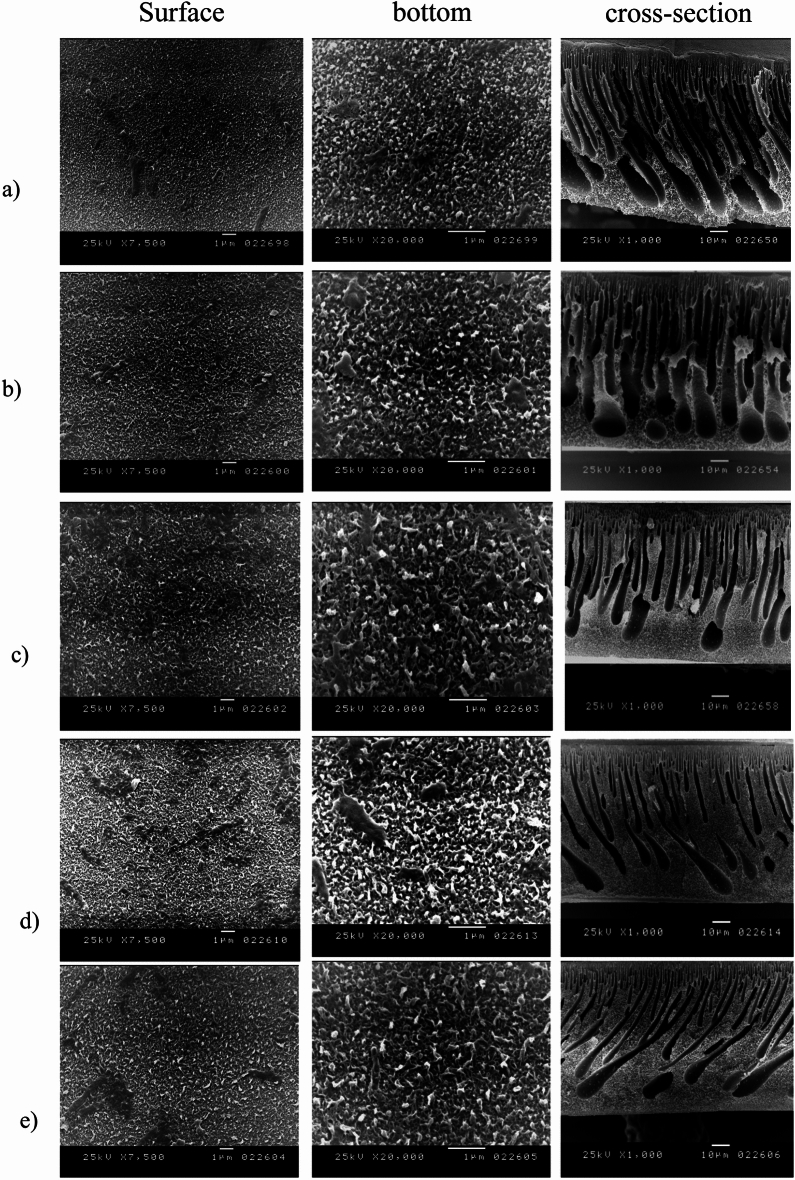
SEM micrographs of TFC-RO membranes fabricated with varying MPD concentrations [(**a**) 0.5 wt%, (**b**) 1 wt%, (**c**) 1.5 wt%, (**d**) 2 wt%, (**e**) 2.5wt%].

In addition to a dense layer near the top surface supporting the active layer, the pore structures feature the necessary finger-like pores extending along the length of the support. As noted by Ghosh et al.^41^, the polyamide active layers in the SEM images exhibit a nodular form. The images highlight the asymmetric structure of the fabricated RO membranes, consisting of a porous sublayer and a dense top layer. The sublayer displays both a macrovoid structure and finger-like cavities. These finger-like cavities in the sublayer result from instantaneous demixing, which is driven by the strong mutual affinity of NMP for water^42^. As seen in Fig. 7, the RO membranes seem to have channels running from top to bottom; at the bottom of the membrane is a sizable hollow, and at the bottom is a fully formed cellular structure. While “macrovoid membranes” are essentially impermeable to water under ultrafiltration conditions, the “nonmacro void” portion of the membranes is made up of a well-developed cellular structure; the membrane with a channel-like shape has a very high permeability to water^43^.

Figures 6a–e illustrates how MPD and TMC concentrations and reaction periods affect the top surface morphology (at two magnifications) and the TFC-RO membrane cross section. The surface morphology of TFC-RO membranes, known as the "ridge-and-valley structure," is nearly identical to that which has been previously documented, according to SEM. On closer inspection, the densely packed globular structure is still visible beneath the ridge-and-valley layer, and it is evident that the protuberance is a direct result of the globules. The SEM analysis of the membrane surface revealed an average pore size of approximately 13nm, with a maximum pore size of 65 nm and a minimum pore size of 7nm. These pore size measurements are critical in determining the membrane’s structural characteristics, directly impacting its filtration efficiency, permeability, and overall performance in separation applications. To allow sufficient MPD to permeate into the organic phase and form these micro-protuberances, the polyamide TFC surface exhibited a layer of densely packed globules and scattered ear-shaped polyamide ridges^44^. These were likely formed on top of PSf regions with surface. A dense layer with smoother surfaces and the well-known “nodular” structure is produced when TMC and MPD concentrations rise^45^.

The PSf support, which serves as a UF membrane, displayed an asymmetric structure with a moderately dense layer on top and a highly porous structure beneath, as shown in the cross-sectional images. Reports indicate that the polyamide active layer formed through interfacial polymerization on the PSf support consists of two distinct layers. The core dense layer is considered to function as the separation barrier in the TFC membrane, forms quickly after the MPD-wetted PSf membrane comes into contact with the organic solution containing TMC. There was abundant MPD at the interface at the start of the interfacial polymerization for condensation polymerization to proceed quickly, followed by polymer chain development and intramolecular and intermolecular crosslinking, until a strongly crosslinked barrier layer was formed^46^.

A relatively open polyamide layer, or the second layer between the core dense layer and the organic phase, forms due to the barrier that limits MPD migration to the organic phase by reducing the polymerization degree and crosslinking density. Since the two layers blend at the boundary and the core dense layer is typically much thinner than the open layer, the asymmetric structure of the two-layer polyamide thin film is challenging to observe in SEM images. The morphology of the cross-section and the film surface appeared unchanged between the control membranes and those treated with alcohol, base, or acid.

### Salt rejection and water flux of TFC-RO membranes

In general, the ultra-thin selective layer’s chemistry and preparation conditions have an impact on the TFC-RO membranes’ performance. Both permeate flux (L/m^2^ h) and salt rejection (%) were measured in order to assess the constructed PA-TFC membranes’ reverse osmosis performance. Cross-flow filtration and an aqueous feed solution with 10,000 ppm NaCl and a pH range of 7 ± 0.2 at 25 °C were used to determine the permeability flux and salt rejection. The applied pressure was 225 psi (50 bar), and the flow rate was 1 g/min. Ten minutes after the cross flow experiment began, all flux and rejection measurements were assessed to make sure the filtration process had stabilized. The permeate flux (J) through a membrane area (A) was calculated as the volume (ΔV) collected during a time period Δt as Eq. (1)^47,49^

1 $${\text{J }} = \, \Delta {\text{V}}/{\text{ A}}. \, \Delta {\text{t}}$$

Also, the salt rejection (R %) was calculated by measuring the electric conductivity of both the feed and permeates solutions using a pH/Conductivity Meter (TDS) (430 portable, Jenway, England) and calculated as follow Eq. (2)^50^

2 $${\text{R}}\% \, = \, \left( {{\text{C}}_{{\text{f}}} - {\text{ C}}_{{\text{p}}} /{\text{C}}_{{\text{f}}} } \right) \, \times { 1}00$$

where C_f_ and C_p_ are the concentrations of the feed and permeate water (product), respectively. Therefore, the study investigates at how preparation parameters affect the production of PA-TFC RO membranes with the optimum RO performance in order to find an ideal set of conditions.

**Fig. 8:**
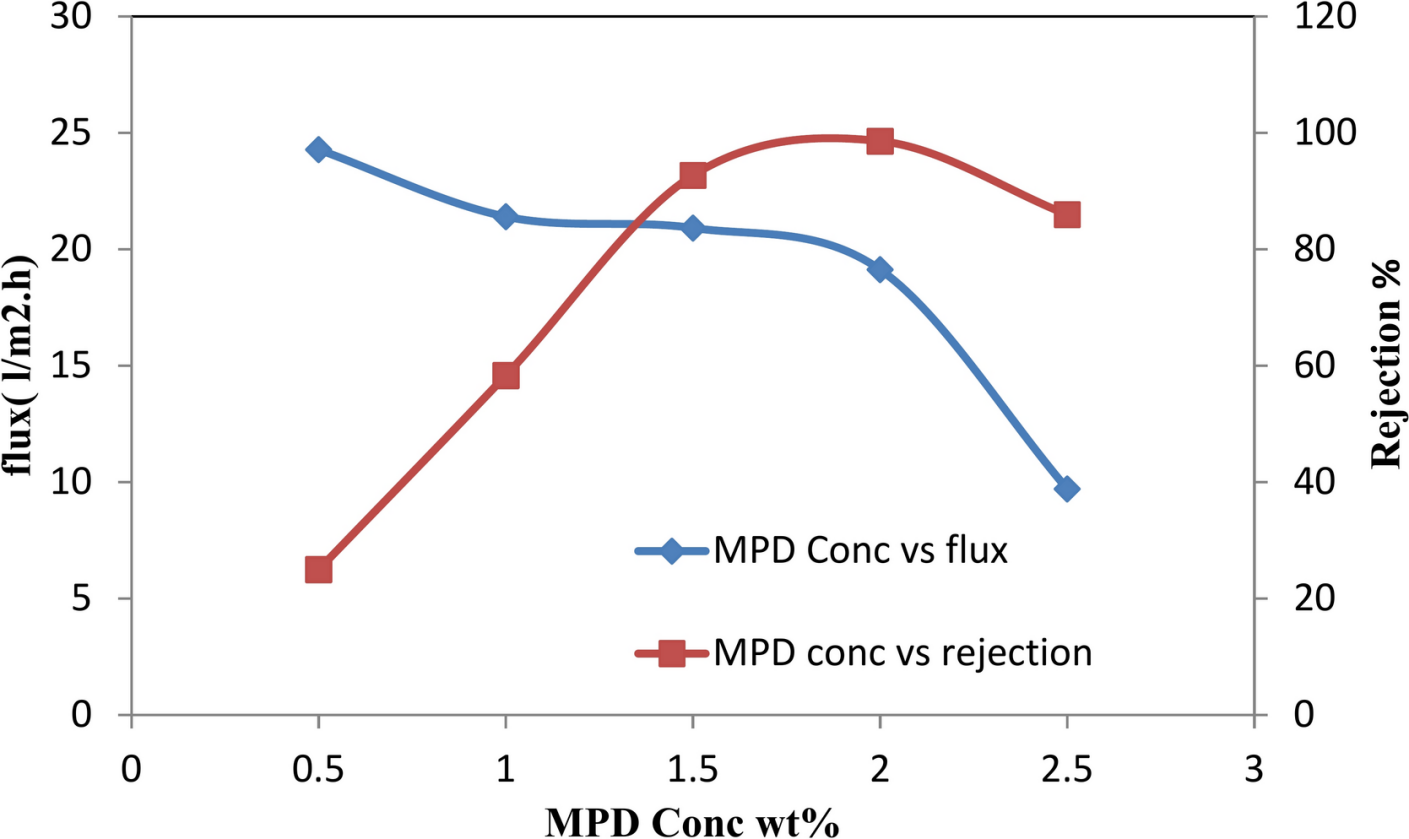
Performance of TFC-RO membranes at varying MPD concentrations with 0.1 w/v% TMC, 1 min reaction time, and 50 bar pressure.

The findings of the experiment showed that higher operating pressures led to higher initial solution flow, suggesting that solution flux and salt rejection are pressure-dependent. Particularly at low ionic strength, the flux drop was larger in solutions with low operating pressures than in those with high operating pressures. Reduced repulsion between the positively charged sodium and the negatively charged membrane led to a decrease in salt rejection at high ionic strength. This, in turn, enhanced the reduction of the double layer thickness at the membrane surface, which in turn decreased salt rejection^51^. The performance of TFC-RO membranes (water flux and rejection) at 50 bars is plotted against MPD concentrations in Fig. 8.

As the MPD concentration increased from 0.5 to 2.5%, the water flux decreased. At higher MPD concentrations, the decline in flux became less pronounced, and at the highest MPD concentration (2.5%), the flux did not reach an asymptotic value. The concentration-sensitive salt rejections rose exponentially at low MPD concentrations, peaked at 2%, and subsequently declined at higher concentrations. Generally speaking, larger layers with higher rejections but lower fluxes are the outcome of high MPD concentration^52^. There is a direct correlation between rejection and film thickness and an inverse relationship between flux and film thickness because this is dependent on the film thickness. As the MPD concentration increases, more MPD molecules are likely to diffuse to the growth front of the film and the aqueous/organic interface. This leads to a higher MPDA/TMC ratio at the interface and an increased likelihood of forming cross-linked units, reducing the amount of linear structure with pendant –COOH groups in the polyamide film. This enhances rejection but reduces flux^53^. Similarly, as TMC concentrations rise from 0.05 to 1.5 wt/v%, water flux decreases. The flux does not reach an asymptotic value at the highest TMC concentration, and the decline in flux becomes more gradual with higher TMC concentrations. Higher TMC concentrations result in more TMC molecules at the organic side of the interface, leading to more unreacted acid chloride groups and, consequently, higher –COOH content in the film. As the results show, decreasing in the flux was not highly influenced by the concentration of TMC. In general high TMC reaction time results in thicker layers with higher rejections but lower fluxes^54,55^. When the reaction time increases, the acid content in the film first quickly decreases to reach a minimum, and then increases steadily. This may be attributed to the “self-limiting” in the reaction process. During the initial stage of polymerization, MPDA molecules diffuse to the organic side of the interface, reacting with TMC to form an initial polyamide film with numerous pendant acid chloride groups. These acid chlorides subsequently react with additional MPDA monomers, creating a denser, more cross-linked film with reduced acid content. As the film grows, MPDA must diffuse through the polyamide layer to reach the organic/film interface, which is slower compared to the diffusion of TMC from the bulk solution to the interface. This imbalance results in excess TMC and the formation of more linear amide units with pendant acid groups. At longer reaction times, the increased film thickness further restricts MPDA diffusion, leading to residual acid groups from the excess TMC. Table 2 presents a comparative analysis of various thin-film composite (TFC) desalination membranes, highlighting differences in material composition, fabrication techniques, rejection efficiency, desalination performance, and water flux.

**Table 2:** A comparative study of desalination membranes, membrane type, material composition, water flux, and test solute.

Membrane type	Material composition	Fabrication technique	Rejection efficiency (%)	Water flux (L/m^2^/h)	Test solute	References
TFC RO	Polyamide	Interfacial polymerization	99.2%	35	2000 ppm NaCl	^56^
PA-G12.5	Modified polyamide	Layer-by-layer deposition	99%	2.5 × 10^−13^	2000 ppm NaCl	^57^
PA/PPESK	*m*-phenylene diamine (MPD) and trimesoyl chloride (TMC)	Interfacial polymerization	99%	22.9	2000 ppm NaCl	^58^
MPD-TMC PMDA/ODA PI	Polyamide + Nanomaterial	Interfacial polymerization	98.5%	3.95	2000 ppm NaCl	^59^
TFC-COF_TpPa-DAPL_	Polyamide TFC RO membrane	Interfacial polymerization	99%	49	2000 ppm NaCl	^60^
M4 membrane	Poly(ether sulfone)	modifying the chemical structure	94.0%	43	2000 ppm NaCl	^61^
TFC RO	Thin film Polyamide	interfacial polymerization	98.8%	30	10,000 ppm NaCl	This work

**Fig. 9:**
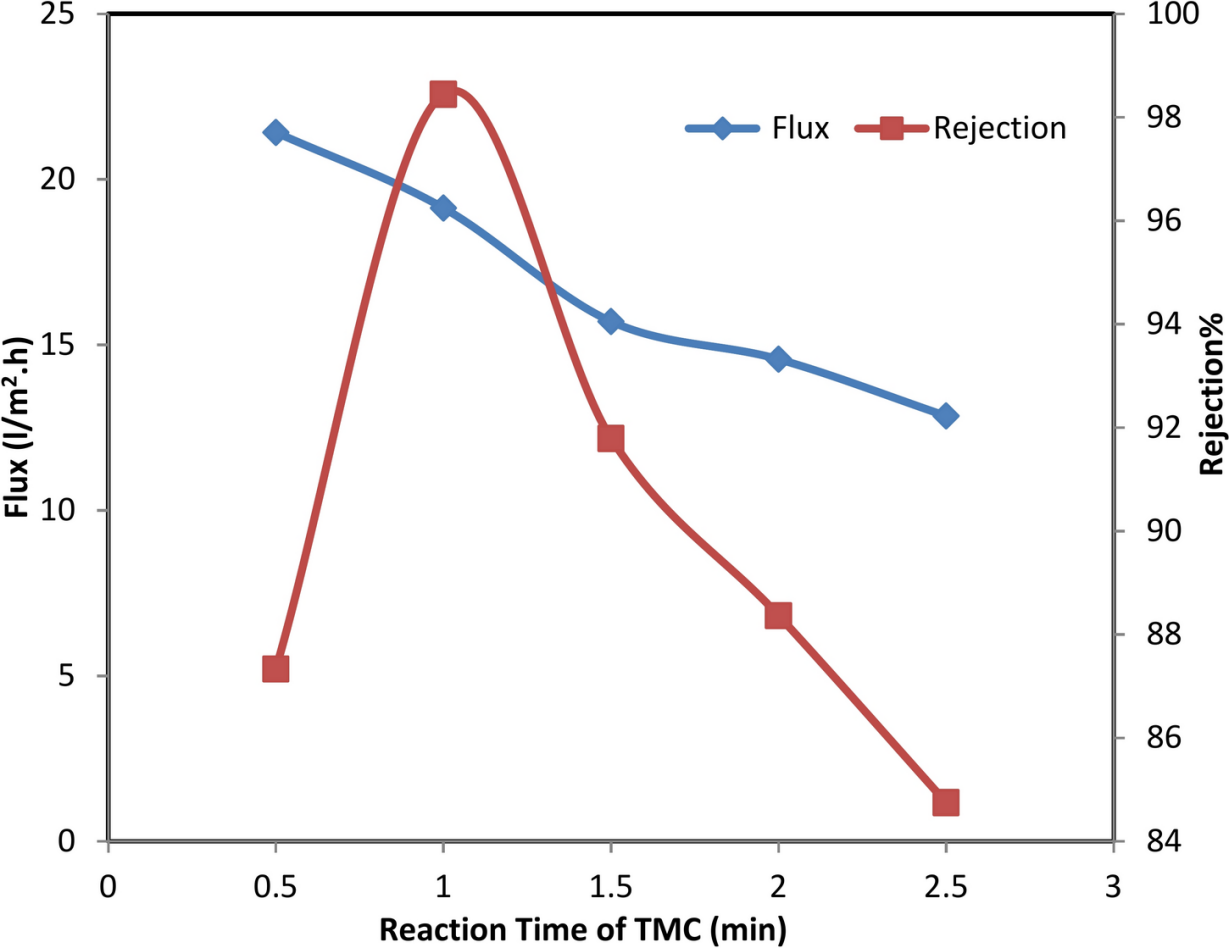
Performance of TFC-RO membranes as a function of the TMC immersion time and TMC concentration was 0.1 w/v% and MPD concentration 2 wt% for 2 min at 50 bar.

The membrane demonstrated stable performance during the first 90 min of testing with a 10,000 ppm NaCl solution at 25 °C and 50 bar (725 psi), maintaining an average water flux of 30 L/m^2^.h and a salt rejection rate of 98.8%. However, after 90 min, the water flux increased from 32 to 58 L/m^2^ h between 90 and 150 min. Simultaneously, salt rejection dropped to 49%, attributed to membrane shrinkage caused by an increase in feed temperature. Figure 9 presents the effect of TMC immersion time during preparation of TFC RO membranes on the salt rejection and water flux. The water flux is decreased as the TMC reaction time was increased from 0.5 to 2.5 min.

## Discussion

The study successfully synthesized and characterized thin-film composite (TFC) membranes for reverse osmosis (RO), demonstrating the impact of monomer concentrations and immersion times on membrane properties. The reduced contact angle (42°–65°) indicated enhanced hydrophilicity compared to the polysulfone (PSf) support layer (91°). Morphological analysis confirmed an asymmetric structure with a dense top layer and a porous sublayer, essential for high performance. The optimized membrane (2 wt% MPD, 0.1 wt/v TMC) achieved 98.6% salt rejection and 19.1 L/m^2^ h water flux. Further research is needed to improve long-term stability, fouling resistance, and scalability for industrial applications.

Evaluating the reusability of membranes is crucial for ensuring their long-term sustainability in desalination and water treatment applications. In this study, the prepared membranes demonstrated stable performance over multiple operational cycles, indicating their initial resistance to fouling and scaling. However, the separation efficiency may decline over time due to the accumulation of contaminants on the surface and pore blockage. To enhance reusability, further research should explore effective chemical and physical cleaning strategies and their impact on the integrity of the polyamide layer. Additionally, surface modifications that reduce organic and inorganic adhesion can significantly improve antifouling properties, extending membrane lifespan and maintaining operational efficiency.

## Conclusion

Thin film composite membranes for reverse osmosis applications were successfully synthesized and characterized. These membranes were based on polyamide and were prepared under different chemical and physical conditions, i.e. monomer concentrations and immersion time. It was observed that the contact angles of the prepared TFC RO membranes have ranged from 42 ± 1 to 65° ± 1. The hydrophilicity of the obtained TFC RO membranes has increased as compared to PSf support layer which has 91° contact angle. The contact angle of the synthesized TFC-RO membranes decreased, indicating increased hydrophilicity, with higher TMC concentrations or lower MPD concentrations. It was observed from scanning electron microscope images that poly sulfone membrane has porous layer that possesses smooth surface and porous bottom while the polyamide thin film composite RO membrane had an asymmetric structure consisting of a dense top layer and a porous sub layer. The sub layers have finger-like cavities as well as macro void structure. The maximum salt rejection and water flux for the prepared thin film composite reverse osmosis membrane were 98.6% and 19.1 L/m^2^ h, respectively obtained at m-phenylene diamine concentration of 2 t% (soaking time for 2 min) and tri mesoyl chloride concentration of 0.1 wt/v reacted for 1 min. This study is an attempt to understand the factors effect of membrane preparation and performance.

### Limitations and future scope

Despite the promising performance of the developed membrane, certain limitations must be addressed to enhance its applicability in real-world scenarios. One key challenge is the potential for membrane shrinkage under high water flux conditions, which may compromise its structural integrity and long-term stability. To mitigate this, future research should focus on structural reinforcement strategies, including the integration of mechanically robust support layers and advanced polymer blends. Additionally, optimizing crosslinking within the polymer matrix and incorporating nanocomposites such as graphene oxide can enhance mechanical durability.

Further advancements in surface hydrophilization techniques, controlled fabrication conditions, and operational parameter optimization can significantly reduce the likelihood of shrinkage while maintaining high permeability. Moreover, scaling up this membrane for industrial applications requires comprehensive evaluations of its performance under varying operational conditions, including prolonged exposure to high-pressure environments and diverse water matrices. Investigating long-term fouling resistance, chemical stability, and energy efficiency will be crucial in determining the membrane’s feasibility for large-scale deployment.

Future studies should also explore the development of hybrid membranes with multifunctional properties, such as enhanced antimicrobial activity and self-cleaning capabilities, to improve operational efficiency and longevity. Implementing these improvements will facilitate the transition of the proposed membrane technology from laboratory-scale research to practical applications in wastewater treatment, desalination, and other high-performance filtration systems.

## Data Availability

The datasets used and/or analyzed during the current study are available from the corresponding author on reasonable request.

## Declarations

### Human and animal participants

This article does not contain any studies involving animals’ studies or human participants performed by any of the authors.
